# Metastatic Medullary Thyroid Carcinoma in Multiple Endocrine Neoplasia Type 2B (MEN 2B) With RET M918T Mutation: Challenges in Long-Term Management and Targeted Therapy

**DOI:** 10.7759/cureus.104392

**Published:** 2026-02-27

**Authors:** Zehra Rahman, Barrie Clark, Kabeer Ali, Hamza Choudhry, Layton Weimer, Kevin Parza, Gabriela Bastidas Mora

**Affiliations:** 1 Internal Medicine, University of Florida College of Medicine – Jacksonville, Jacksonville, USA; 2 Hematology and Medical Oncology, University of Florida College of Medicine – Jacksonville, Jacksonville, USA

**Keywords:** adrenal pheochromocytoma, malignant thyroid nodule, medullary thyroid carcinoma, multiple endocrine neoplasia type 2b (men2b), ret kinase inhibitor

## Abstract

Multiple endocrine neoplasia type IIB (MEN2B) is a rare hereditary cancer syndrome characterized by medullary thyroid carcinoma (MTC), pheochromocytoma, and distinctive mucosal neuromas. MEN2B-associated MTC is most often caused by *RET* M918T mutations and confers an earlier and more aggressive disease progression. The aggressive nature of *RET* M918T-mutated MEN2B emphasizes the necessity of vigilant lifelong surveillance. It also highlights the real-world challenges of maintaining continuity of targeted therapy, where treatment interruptions may compromise disease control. This reports the case of a 25-year-old woman diagnosed with MTC in the setting of MEN2B. Initial evaluation revealed extensive metastases involving the regional neck lymph nodes, lungs, and adrenal glands. She underwent total thyroidectomy with bilateral neck dissection. Genetic testing confirmed the pathogenic *RET* M918T mutation. Family screening was negative for *RET* mutations in her mother and brother; her father, who died at 37 from uncontrolled hypertension, may have had an undiagnosed pheochromocytoma. Two years postoperatively, biochemical surveillance detected elevated plasma metanephrines, and subsequent workup confirmed bilateral pheochromocytomas. She underwent staged adrenal-sparing surgeries. Given progressive metastatic disease, selpercatinib therapy was initiated but required dose adjustments due to gastrointestinal intolerance. Treatment interruptions occurred secondary to funding and follow-up challenges. Upon re-evaluation one year later, imaging revealed recurrent paratracheal, pulmonary, hepatic, and possible adrenal metastases, prompting re-initiation of selpercatinib at a reduced dose, which she tolerated and continues to this day with surveillance of symptoms, serial electrocardiograms, laboratory work, and imaging. This case illustrates the aggressive course of *RET* M918T-mutated MEN2B and underscores the importance of early genetic diagnosis, vigilant surveillance, and continuity of selective RET inhibitor therapy to optimize disease control.

## Introduction

Medullary thyroid carcinoma (MTC) is a rare neuroendocrine tumor that arises from the parafollicular C cells of the thyroid gland and accounts for approximately 1-2% of all thyroid cancers [1,2]. The pathogenesis of the tumor results from persistent activation of intracellular signaling pathways, most commonly through activating mutations in the *RET* proto-oncogene or, less frequently, somatic mutations in the RAS signaling pathway. Approximately 75% of cases are sporadic, whereas 20-25% occur as part of hereditary cancer syndromes caused by germline *RET* mutations [2-4]. These genetic alterations lead to C-cell hyperplasia and excessive production of calcitonin and carcinoembryonic antigen (CEA), which serve as important diagnostic and prognostic biomarkers [2,5].

Hereditary MTC occurs in association with multiple endocrine neoplasia type 2 (MEN2), an autosomal dominant syndrome with two major subtypes: MEN2A and MEN2B, as well as familial medullary thyroid carcinoma (FMTC). MEN2A is the most common subtype and typically presents in adolescence or adulthood. It is characterized by MTC, pheochromocytoma, and primary hyperparathyroidism due to parathyroid hyperplasia. In contrast, MEN2B is exceedingly rare, with an estimated prevalence ranging from one in 600,000 to one in four million, and is characterized by early-onset, aggressive MTC, pheochromocytoma, mucosal neuromas, intestinal ganglioneuromatosis, and a marfanoid body habitus [1]. Unlike MEN2A, MEN2B is not associated with parathyroid disease and often presents in childhood with rapidly progressive metastatic disease. MEN2B is most commonly associated with the *RET* M918T mutation, which confers a particularly poor prognosis because of its aggressive clinical behavior and early onset of metastatic disease [6].

Prognosis in MTC varies significantly based on disease stage, genetic profile, and treatment approach; prognosis is often poor due to delayed diagnosis and the tendency towards metastatic disease [2,7]. Patients with localized disease treated with curative surgical resection have favorable outcomes, with reported 10-year overall survival rates of 75-90%. However, survival decreases substantially in advanced disease, with 10-year survival rates of approximately 40-70% for regional disease and 20-40% for distant metastatic disease [8]. For early-stage disease, total thyroidectomy with central lymph node dissection is the standard of care, with lateral neck dissection considered in more extensive cases. In patients with unresectable, recurrent, or metastatic disease, systemic therapy is indicated. Historically, management has relied on multikinase inhibitors such as vandetanib and cabozantinib, which target RET along with other signaling pathways. These agents improve progression-free survival but are limited by off-target toxicities, including hypertension, diarrhea, QT prolongation, fatigue, and dermatologic adverse effects [9].

More recently, highly selective RET inhibitors such as selpercatinib have emerged as preferred options for *RET*-mutant MTC due to improved response rates and tolerability compared with older multikinase inhibitors. Selpercatinib is a highly selective RET tyrosine kinase inhibitor whose activity against *RET* M918T makes it particularly relevant in aggressive MEN2B-associated disease [10]. Selpercatinib selectively inhibits RET signaling, including the *RET* M918T mutation commonly observed in MEN2B, and has demonstrated objective response rates exceeding 60-70% in advanced disease, with durable responses and improved safety profiles compared with earlier therapies [11]. Common adverse effects of selpercatinib include hypertension, elevated liver transaminases, diarrhea, fatigue, edema, rash, constipation, dry mouth, and QT interval prolongation. Serum calcitonin and CEA remain standard biomarkers for disease monitoring; however, detection of circulating cell-free DNA harboring *RET* mutations has been associated with worse overall survival and may serve as a sensitive marker of disease progression [12].

We present a case of metastatic MTC in a young woman with MEN2B due to a *RET* M918T mutation who subsequently developed bilateral pheochromocytomas and required treatment with the selective RET inhibitor selpercatinib. This case illustrates the aggressive nature of MEN2B-associated MTC and highlights the importance of early diagnosis and the expanding role of targeted therapy.

## Case presentation

A 25-year-old woman presented to the Hematology/Oncology clinic in 2025 for a follow-up consultation on MEN2B syndrome, which was diagnosed 11 years prior.

She was first evaluated at age 14 (November, 2014) for bilateral thyroid nodules, and fine-needle aspiration of a left nodule was consistent with MTC. She subsequently underwent total thyroidectomy with bilateral central and modified radical neck dissections. Pathology revealed bilateral medullary thyroid carcinoma and C-cell hyperplasia. The largest tumor size was three cm on the right with negative margins and positive extrathyroidal extension. Ten out of 64 lymph nodes were involved with disease, and there was positive extranodal extension. The disease was staged as pT3N1bM0 (American Joint Committee on Cancer (AJCC) Cancer Staging Manual, 7th Edition [13]), Stage IVA. Genetic testing performed in December of 2014 identified a germline *RET* gene mutation, p.M918T, confirming the diagnosis of MEN2B. Clinically, she exhibited a MEN2B phenotype characterized by marfanoid habitus, pes cavus, oral mucosal neuromas, absence of tears, a history of frenulectomy, and megacolon. Family screening revealed no *RET* mutations in her mother or brother. Her father, who had died at age 37 from uncontrolled hypertension with left ventricular hypertrophy, was suspected to have had an undiagnosed pheochromocytoma.

Staging imaging two years later, at age 16 (2016), demonstrated metastatic disease involving regional cervical lymph nodes and bilateral adrenal masses. Initial biochemical evaluation demonstrated plasma metanephrines, and metaiodobenzylguanidine (MIBG) imaging showed bilateral adrenal uptake (right greater than left), confirming bilateral pheochromocytomas. She underwent a cortical-sparing right posterior retroperitoneoscopic adrenalectomy. Pathology confirmed right-sided pheochromocytomas measuring 1.9 × 1.8 × 1.1 cm and 2.0 × 1.3 × 0.7 cm. She subsequently underwent staged cortical-sparing surgery for a left adrenal pheochromocytoma. Her labs and subsequent imaging at this time showed stable disease activity. After surgical interventions, the patient had a prolonged period of time during which she had intermittent follow-up with minimal disease surveillance due to financial difficulties. She returned for consistent follow-up seven years later (2023), where she was demonstrated to have biochemical and radiologic evidence of persistent and progressive disease. Her calcitonin levels rose from 172 pg/mL to 3,166 pg/mL (reference ≤5 pg/mL), and CEA increased from 15.8 ng/mL to 92.4 ng/mL (reference 0.0-3.0 ng/mL) as shown in Table 1 in the seven-year time span without adequate follow-up. Imaging at this time revealed stable bilateral thyroid bed nodules, multiple indeterminate pulmonary nodules, and small hypervascular hepatic lesions concerning for metastatic disease.

**Table 1:** Pertinent laboratory analysis throughout the clinical course. CEA: carcinoembryonic antigen; TSH: thyroid-stimulating hormone; T4: thyroxine

Laboratory Parameter	Reference Range	Measured Value, 2014	Measured Value, 2016	Measured Value, 2023	Measured Value, March 2025	Measured Value, November 2025 (after selpercatinib reinitiation)
Plasma Metanephrines	<25 pg/mL	168	2.1	4.9	138	<25
Plasma Normetanephrines	0-210 pg/mL	230	3.0	6.7	324.6	77.7
Calcitonin	0-5 pg/mL	1722	172.0	3166	3617	73.7
CEA	0-3.8 ng/mL	66.7	15.8	92.4	147	27.1
TSH	0.270-4.200 mIU/L	2.84	8.51	1.98	0.920	3.60
T4	0.80-1.70 ng/dL	1.3	1.73	1.74	1.52	1.95

Given progressive metastatic MTC, she was initiated on selpercatinib 120 mg twice daily in 2023. At the time of treatment initiation in 2023, the patient demonstrated preserved functional capacity with independence in activities of daily living and no significant physical limitations. Although a formal performance scale such as the Karnofsky Performance Status was not recorded, her clinical condition was consistent with good performance status and eligibility for systemic therapy. The dose of selpercatinib was reduced within one year to 80 mg twice daily due to gastrointestinal intolerance, primarily uncontrolled diarrhea, which was interfering with her normal daily living activities.

There was subsequently a pause in treatment due to financial and follow-up barriers; the patient reported she ran out of the medication in July of 2024. Upon return to follow-up in March of 2025, imaging revealed recurrent enhancing nodules in the paratracheal soft tissues, multiple subcentimeter pulmonary nodules, and hypervascular liver lesions consistent with metastatic recurrence. A bone scan revealed no evidence of osseous metastatic disease. A 1.1 × 0.8 cm soft tissue nodule was also identified at the site of the right adrenal gland, though its significance remained uncertain given prior surgery. Plasma metanephrines remained below the reference range. Selpercatinib therapy was reinitiated at the reduced dose of 80 mg twice daily at this follow-up visit in March 2025. At a serial follow-up eight months after initiation of selpercatinib in November of 2025, her calcitonin level had fallen to 73.7 pg/mL, and her CEA was 27.1 ng/mL, consistent with a favorable biochemical response.

Pertinent laboratory findings throughout the patient’s clinical course are summarized in Table 1. Her post-surgical hypothyroidism has been well-managed by endocrinology. Since restarting selpercatinib, repeat CT imaging demonstrated stability of subcentimeter pulmonary micronodules, a reduction in the size of liver lesions, and unchanged size of right adrenal gland soft tissue nodule, indicating radiographic disease control. 

Patients receiving selpercatinib require regular monitoring for treatment-related toxicities and disease progression. Accordingly, the patient underwent serial blood pressure monitoring due to the risk of treatment-associated hypertension and periodic electrocardiograms to evaluate for QT interval prolongation, a known adverse effect of RET inhibition. Plasma-free metanephrines were assessed to monitor for biochemical evidence of pheochromocytoma recurrence in the setting of MEN2-associated disease. Surveillance imaging with CT of the chest, abdomen, and pelvis was performed to assess treatment response, while laboratory monitoring included complete blood count and comprehensive metabolic panel to evaluate for hematologic or hepatic toxicity, along with serial calcitonin and CEA measurements to track tumor burden.

**Figure 1:**
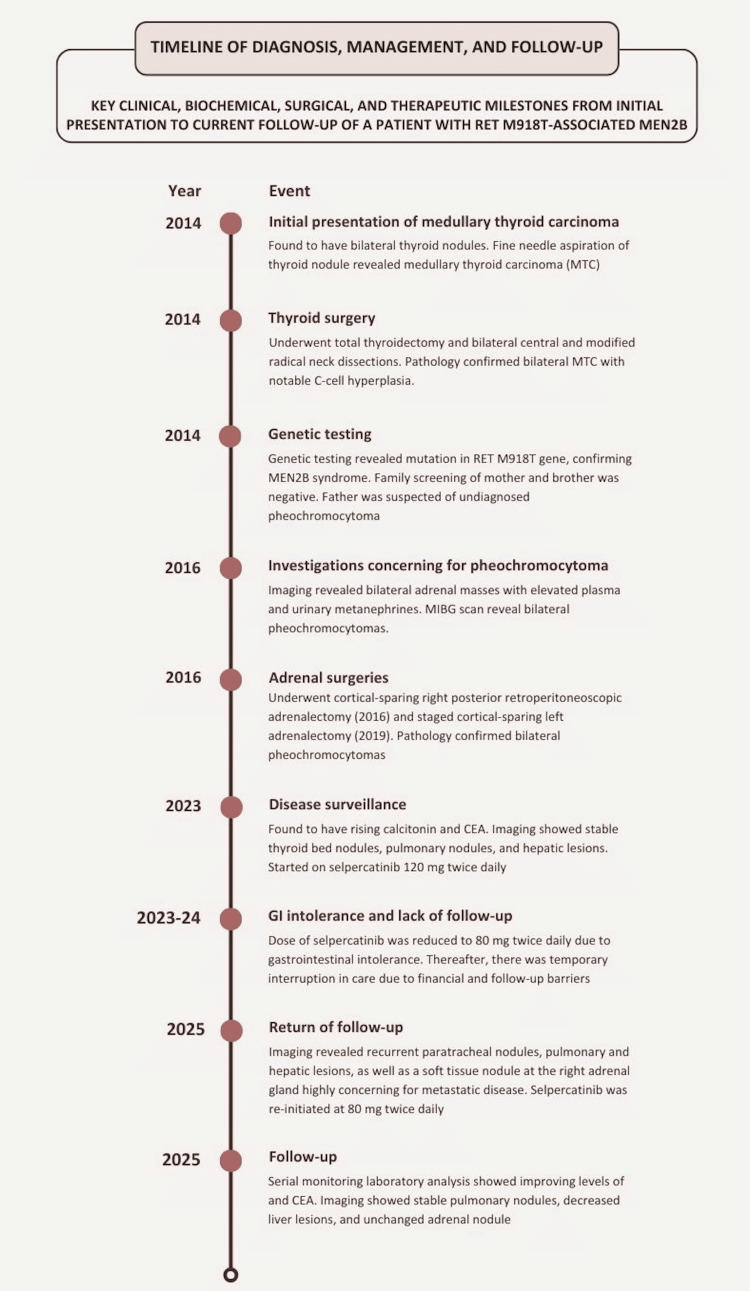
Timeline of key diagnostic, surgical, biochemical, and therapeutic events Image created with Canva (https://www.canva.com)

In patients with metastatic MTC, selective RET inhibitors such as selpercatinib are generally continued indefinitely until disease progression or unacceptable toxicity occurs. Dose interruption, reduction, or discontinuation may be considered in cases of significant adverse effects, whereas treatment cessation may be appropriate in the setting of sustained intolerance or progression despite therapy. In this patient, therapy has been well tolerated with continued clinical benefit, and therefore, treatment was maintained with close longitudinal monitoring. A detailed timeline of the patient’s diagnostic evaluation, disease progression, and management milestones is depicted in Figure 1. 

## Discussion

MTC in the context of MEN2B represents one of the most aggressive forms of hereditary thyroid malignancy [14]. MEN2B is caused primarily by the *RET *M918T mutation, which leads to constitutive activation of the RET tyrosine kinase receptor and uncontrolled downstream signaling. This specific mutation is associated with early disease onset, rapid progression, and a higher likelihood of distant metastases at diagnosis compared with other *RET* variants. This patient’s presentation with disseminated disease at age 25 exemplifies this aggressive clinical phenotype.

The detection of the *RET* M918T mutation has diagnostic, prognostic, and therapeutic implications. While calcitonin remains the traditional biomarker for MTC disease burden, recent studies suggest that circulating cell-free *RET* M918T DNA may correlate more closely with disease progression and overall survival [15]. This molecular marker may provide a more sensitive indicator of treatment response and early recurrence, particularly in patients receiving targeted therapy.

Management of MTC associated with MEN2B requires a multidisciplinary approach involving endocrinology, oncology, surgery, and genetics. Early prophylactic thyroidectomy during infancy is recommended in known mutation carriers to prevent early metastatic spread [16]. However, as in this case, delayed diagnosis often results in advanced disease at presentation. Total thyroidectomy with central and lateral neck dissection remains the cornerstone of treatment for disease confined to the thyroid and regional lymph nodes, but curative surgery is rarely achievable in metastatic cases [17].

The development of RET-specific tyrosine kinase inhibitors such as selpercatinib and pralsetinib has transformed the therapeutic landscape for advanced or metastatic MTC. In the long-term follow-up of the phase I/II LIBRETTO-001 trial, selpercatinib achieved an objective response rate (ORR) of approximately 82.5% in treatment-naïve *RET*-mutant MTC and 77.6% in previously treated MTC, with median progression-free survival (PFS) not reached in the naïve cohort and 41.4 months in the pretreated cohort [14]. Furthermore, in the phase III LIBRETTO-531 randomized study in *RET*-mutant MTC, selpercatinib significantly prolonged PFS compared with cabozantinib or vandetanib (hazard ratio ≈ 0.28) at 12-month follow-up [10]. These agents selectively inhibit RET-driven signaling, leading to meaningful and durable clinical responses with improved tolerability compared with earlier multikinase inhibitors such as vandetanib or cabozantinib [10,18]. 

Selpercatinib and pralsetinib are approved in multiple regions, including the United States, the European Union, and Japan. However, access varies across healthcare systems due to differences in regulatory approval timelines, cost, insurance coverage, and reimbursement policies. Although these therapies are generally available in the United States, high costs and insurance barriers may limit access, whereas other countries may offer broader reimbursement or experience delays related to resource constraints [19]. In this patient, selpercatinib achieved disease control but required dose adjustment for gastrointestinal side effects and was briefly interrupted due to funding issues, highlighting the practical challenges of maintaining targeted therapy in rare cancers. Such interruptions can contribute to disease progression, underscoring the need for consistent access and adherence support for optimal outcomes.

Peptide receptor radionuclide therapy (PRRT) represents an emerging treatment modality for metastatic MTC with documented somatostatin receptor expression and may be considered in progressive disease or intolerance to systemic targeted therapy. However, its role remains limited and is generally reserved for selected cases. In this patient, PRRT was not pursued, given an adequate clinical response to selpercatinib [20].

The patient’s subsequent development of bilateral pheochromocytomas is consistent with the MEN2B phenotype. Lifelong biochemical surveillance for catecholamine-secreting tumors is crucial in this population, as undiagnosed pheochromocytoma can lead to hypertensive crises, as possibly occurred in this patient’s father. Adrenal-sparing surgery, when feasible, remains the preferred approach to preserve cortical function and avoid lifelong steroid dependence [21].

This case reinforces the aggressive natural history of *RET* M918T-mutated MEN2B and illustrates the critical importance of early genetic testing, family screening, and multidisciplinary surveillance. Advances in RET-targeted therapies have significantly improved outcomes for patients with metastatic disease, but long-term management remains complex due to drug intolerance, socioeconomic barriers, and the need for continuous monitoring for recurrent or progressive disease.

## Conclusions

This case underscores the aggressive nature of MTC associated with the *RET *M918T mutation in MEN2B and highlights the need for early recognition, genetic counseling, and lifelong surveillance. Targeted RET inhibition with selpercatinib offers meaningful and durable disease control in advanced disease, though treatment continuity and access remain critical challenges. Close multidisciplinary coordination among endocrinology, oncology, surgery, and genetics teams is essential to optimize patient outcomes and long-term management in this rare hereditary cancer syndrome.
